# Association between lifestyle factors and hypertension control in Indonesian primary healthcare settings: A cross-sectional study

**DOI:** 10.51866/oa.409

**Published:** 2024-06-12

**Authors:** Ermin Rachmawati, Riskiyah, Qanita Adzkia Novindra, Nadia Alfi Syarifah, Nahda Rihadatul Aisy

**Affiliations:** 1 MD, M.Biomed, Dr, Department of Biomedical Sciences, Faculty of Medicine and Health Sciences UIN Maulana Malik Ibrahim, Malang, Jl. Locari, Tlekung, Kec., Junrejo, Batu East Jawa, Indonesia. Email: ermin.rachmawati@kedokteran.uin-malang.ac.id; 2 MD, MMRS, Department of Public Health, Faculty of Medicine and Health Sciences UIN Maulana Malik Ibrahim, Malang, Jl. Locari, Tlekung, Kec., Junrejo, Batu East Jawa, Indonesia.; 3 Medical Profession Program, Faculty of Medicine and Health Sciences UIN Maulana Malik Ibrahim, Malang, Jl. Locari, Tlekung, Kec., Junrejo, Batu East Jawa, Indonesia.; 4 Medical Profession Program, Faculty of Medicine and Health Sciences UIN Maulana Malik Ibrahim, Malang, Jl. Locari, Tlekung, Kec., Junrejo, Batu East Jawa, Indonesia.; 5 Medical Profession Program, Faculty of Medicine and Health Sciences UIN Maulana Malik Ibrahim, Malang, Jl. Locari, Tlekung, Kec., Junrejo, Batu East Jawa, Indonesia.

**Keywords:** Hypertension, Lifestyle, Physical activity, Sleep quality, Subjective stress

## Abstract

**Introduction:**

A healthy lifestyle influences hypertension control. However, studies investigating the effects of lifestyle on hypertension remain elusive. This study aimed to analyse the association between lifestyle factors and hypertension control among patients with hypertension.

**Methods:**

This cross-sectional exploratory study was conducted from June to December 2022 among 265 patients with hypertension from the Pusat Kesehatan Masyarakat. The status of hypertension control was assessed by checking the serial blood pressure. The physical activity (PA) level, sleep quality, stress level and eating pattern were measured using the Global Physical Activity Questionnaire; Pittsburgh Sleep Quality Index; Depression Anxiety Stress Scale-21; and 24-Hour Food Recall Questionnaire, Adolescent Food Habits Checklist and Emotional Eating Scale, respectively. Stepwise binary logistic regression and a generalised linear model were used for the statistical analysis.

**Results:**

Approximately 72.2% of the participants had uncontrolled hypertension. The majority showed a low PA level (46%), normal stress level (94.7%), good sleep quality (80%), low caloric intake (95.5%), neutral food habit (55.5%) and low emotional eating (93.2%). Sex (P=0.030), age (P=0.018), PA level (P=0.011), sleep quality (P=0.032) and stress level (P=0.030) significantly influenced hypertension control. Specifically, moderate (odds ratio [OR]=5.868, 95% confidence interval [CI]=3.024-11.798, P=0.000) and vigorous PA levels (OR=2.188, 95% CI=1.026-4.678, P=0.042) were significantly associated with hypertension control.

**Conclusion:**

Moderate and vigorous PA levels are lifestyle factors that may play a role in controlling hypertension.

## Introduction

Hypertension remains the leading cause of cardiovascular disease (CVD) worldwide.^1^ The increasing number of cardiovascular complications and deaths caused by hypertension might be explained by uncontrolled hypertension among patients.^2^ In 2011, the World Health Organization (WHO) stated that nearly 1 billion individuals were living with poorly controlled hypertension worldwide.^3^ The prevalence of uncontrolled hypertension in developed and developing countries is 66.8% and 61.6%, respectively.^4^

Several factors contribute to uncontrolled hypertension in patients, including genetic predisposition, lifestyle, comorbidity, attitude towards accessing health services and medication adherence. Compared with other factors, lifestyle plays a predominant role in controlling hypertension, being the first intervention recommended for all stages of the disease.^4-6^ Animut et al. reported that adult patients with hypertension who routinely performed moderate physical activity (PA), consumed vegetables and limited their salt consumption had a twofold higher prevalence of controlled hypertension.^7^ The study by Hu et al. among middle-aged female patients with hypertension in China showed an increased blood pressure (BP) with high psychological stress levels.^8^ Poor subjective sleep quality is significantly associated with higher BP. Good sleep quality can help reduce BP and better manage hypertension.^9^ Caloric restriction also decreases BP, with an average of 90% of patients achieving a BP of <140/90 mmHg.^10^

A previous national survey conducted in Indonesia provided data on hypertension prevalence but not on hypertension control status. Currently, the Indonesian Ministry of Health’s priority programme for managing hypertension focuses on primary prevention and the use of antihypertensive drugs. Although the programme has already included lifestyle interventions for patients with hypertension, its effectiveness is yet to be determined.^11^ Therefore, this study aimed to analyse the lifestyle factors associated with hypertension control using an exploratory research approach, which could be used to create a valid predictive model in the future. This study was a representative survey assessing BP control as a way of gauging the effectiveness of the health programme in managing hypertension. Hence, the results could provide a more comprehensive overview of the problems encountered in managing uncontrolled hypertension and be used as a reference for formulating policies and redesigning the programme.

## Methods

### Study design and participant selection

All study protocols were approved by the Ethical Committee of FKIK UIN Maulana Malik Ibrahim Malang under registration number 103/EC/KEPK-FKIK/2022. This study was conducted at the Pusat Kesehatan Masyarakat (Puskesmas), a primary healthcare facility commonly found in Indonesia, which has an essential role in delivering basic healthcare services to the community at the grassroot level and providing information needed to develop health policies.

The Lemeshow formula (total population: 10,110; prevalence of uncontrolled hypertension: 61.6%) was used to calculate the sample size. Participants were selected using convenience sampling: Members of the target population met certain practical criteria such as easy accessibility, geographical proximity and availability at a given time. Prior to participant selection, the researchers conducted briefings with health centre staff (doctors and nurses) regarding the inclusion and exclusion criteria for participants.

Outpatients who visited the Puskesmas from June to December 2022 were included as study participants when they met two prerequisites: (1) diagnosis of hypertension from the doctors based on medical records and (2) willingness to participate in the study. Patients who were unable to read and write, had mental disorders or memory problems, follow diet program, limited mobility due to musculoskeletal or metabolic disorder, or did not complete the questionnaire were not allowed to participate.

### Sociodemographic and clinical variables

The sociodemographic and clinical variables measured consisted of age, sex, educational attainment, employment history, family income and the presence of comorbidities. The Indonesian Ministry of Health’s age classification criteria were applied, while Badan Pusat Statistik Indonesia’s recommendations were used for the classification of family income and educational attainment. Occupation was categorised according to the authors’ preference based on participants’ answers.

### Hypertension control

The two categories of hypertension control status—uncontrolled (BP of ≥140/90 mmHg) and controlled (BP of <140/90 mmHg)—were established by averaging three serial data points of BP: two from medical records and one from a direct measurement taken at the current appointment.

### PA level

The PA level was assessed using 16 questions of the Global Physical Activity Questionnaire divided into three parts: components of PA at work, travel activities from one place to another and recreational or free-time activities carried out in a week.^12^ In the questionnaire, the result is calculated and interpretated as follows: (1) vigorous when (P2+P11) is ≥3 days, and the total metabolic equivalent (MET) minutes per week is ≥1500, or when (P2+P5+P8+P11+P14) is ≥7 days, and the total MET minutes per week is ≥3000; (2) moderate when (P2+P11) is ≥3 days, and ((P2xP3)+(P11xP12)) is ≥60 min; when (P5+P8+P14) is ≥5 days, and ((P5xP6)+(P8xP9)+(P14xP15) is ≥150 min; or when (P2+P5+P8+P11+P14) is ≥5 days, and the total MET minutes per week is 600–3000; and (3) low when the MET value is <600, or the MET value does not meet the criteria for moderate or high levels of PA.

### Sleep quality

The Pittsburgh Sleep Quality Index was used to determine sleep quality. This instrument comprises seven areas: sleep latency, subjective sleep quality, daily sleep efficiency, sleep duration, sleep disruptions, usage of sleeping medicines and dysfunction throughout the day. The total possible score ranges from 0 to 21. The evaluation criteria are divided into two categories: poor sleep quality and good sleep quality, depending on whether the total value is ≥5.^13^

### Stress level

This study used the Indonesian version of the Depression Anxiety Stress Scale (DASS)- 21, which is an adaptation of the DASS-42 and composed of 21 questions. This questionnaire consists of three scales, namely depression, anxiety and stress. Each scale consists of seven questions and corresponds to an answer ranging from 0 to 3: 0=never at all, 1=sometimes, 2=often and 3=very often. The total score is interpreted as normal, mild, moderate, severe and extremely severe stress levels.^14^

### Caloric intake

Caloric intake was calculated using the 24-Hour Food Recall Questionnaire. This protocol and interpretation adopted the latest regulation of the Indonesian Ministry of Health. Briefly, respondents are asked to recall the food consumed in the last 24 h and then fill out the questionnaire. The results are interpreted as a percentage of the value of nutritional adequacy (AKG) using Nutrisurvey. The nutritional adequacy of caloric intake is then categorised into three criteria: low, normal and excessive (<90%, 90%–120% and >120% AKG, respectively).

### Food habits

The Adolescent Food Habits Checklist (AFHC) includes 23 statements answerable by ‘yes’ or ‘no’. In nine statements, there is a choice indicating that the statement does not apply to respondents. One point is scored when respondents have a healthy eating habit response (answer ‘no’ to statements 3, 8, 14, 18 and 21 and ‘yes’ to the rest of the statements). The final score is adjusted to the response that is not filled out and does not apply (statements 1, 6, 7, 11, 17, 18, 19, 20 and 21) using the following formula: AFHC score=number of responses to healthy eating habitsx(23/number of items completed). The AFHC score is divided into four quartiles: The first quartile indicates unhealthy eating habits (scores 0–5); second and third quartiles, neutral eating habits; and fourth quartile, healthy eating habits (scores 18–23).^15^

### Emotional eating

The Emotional Eating Scale comprises 25 selfreported items. It assesses the urge to eat when experiencing negative emotions such as anger, anxiety and poor mood states (depression). The items are scored on a 5-point Likert scale, ranging from 0 (no desire to eat) to 4 (extreme desire to eat). Higher scores indicate a dependence on eating food to help control emotions. The total score is determined by summing the scores of all items (0–100). A score of 25 indicates low emotional eating (substantially healthy relationship with food); 26–46, moderate emotional eating; and ≥47, high emotional eating.^16^

### Data analysis

The IBM SPSS Statistics for Windows, version 26 (IBM Corp., Armonk, N.Y., USA), was used for the data analysis. The sociodemographic and lifestyle factors were presented as frequencies. Stepwise binary logistic regression was employed to examine the association of the lifestyle factors with hypertension control and develop a predictive model. The final multivariable binary logistic regression model was found to fit based on the findings of the Hosmer–Lemeshow goodness-of-fit test. The magnitude of the association between the predictor and response variables was measured based on odds ratios (ORs) and 95% confidence intervals (CIs) using a generalised linear model. P-values of <0.05 were considered statistically significant.

## Results

### Sociodemographic and clinical characteristics

**Figure 1:**
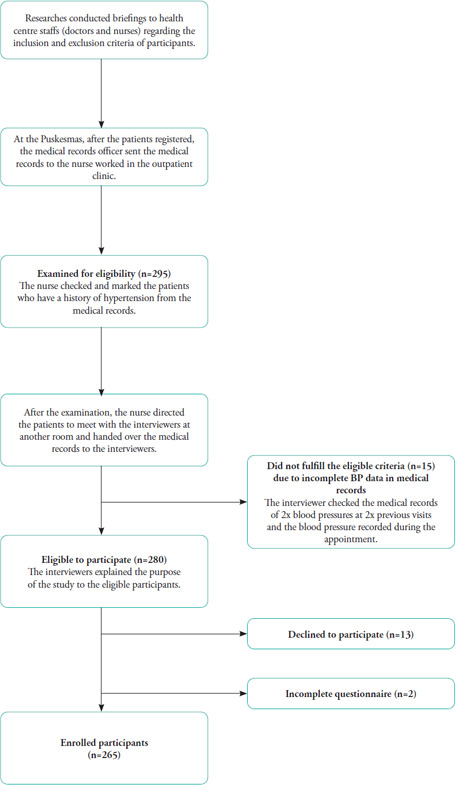
Flowchart of participant selection.

A total of 295 patients with hypertension visited the Puskesmas from June to December 2022. Potential eligible participants were determined based on the presence of a history of hypertension in the medical record screened by nurses and doctors on patients’ arrival at the outpatient clinic in the Puskesmas. Interviewers checked the BP in the medical records at two previous visits, and BP was recorded during the current appointment in a different room after the consultation and examination sessions. Thirty patients were excluded because of the following reasons: incomplete BP data (n=15), refusal to participate after receiving an explanation about the purpose of the study (n=13) and noncompletion of the questionnaire (n=2). The minimum number of participants calculated was 351. However, only 265 participants were ultimately included in this study (Figure 1).

The profiles of the 265 participants are displayed in Table 1. The participants were predominantly women (76.6%) and early older adults (aged 56–65 years) (59.6%). Many participants graduated from elementary school only (48.7%). The majority did not have an occupation (50.2%) and had a family income of <1 million rupiah (58.9%).

**Table 1:** Sociodemographic and clinical profiles of the participants.

Characteristics	n	%
**Sex**		
Female	203	76.6
Male	62	23.4
**Age, year**		
36-45	8	3
46-55	71	26.8
56-65	158	59.6
>66	28	10.6
**Comorbidity**		
None	106	40
Diabetes mellitus	77	29.1
Hyperlipidaemia	30	11.3
Hyperuricaemia	20	7.5
Cerebrovascular accident	10	3.8
Coronary heart disease	14	5.3
Kidney disease	1	0.4
Hypertensive heart disease	2	0.8
Congestive heart failure	5	1.9
**Educational attainment**		
No education	15	5.6
Elementary school	129	48.7
Junior high school	55	20.8
Senior high school	42	15.8
Diploma	8	3
Bachelor	16	6
**Profession**		
Civil servant	4	1.5
Private employee	7	2.6
Self-employed	23	8.7
Farmer	24	9.1
Fisherman	2	0.8
Labourer	14	5.3
Unemployed	133	50.2
Pensioner	58	21.9
**Family income**		
Low (<1 million)	156	58.9
Middle (1.5–2.5 million)	82	30.9
High (2.5–3.5 million)	20	7.5
Very high (>3.5 million)	7	2.6

### Hypertension control status

The hypertension control status and medication intake among the participants are presented in Table 2. A total of 193 (72.2%) participants had uncontrolled hypertension.

**Table 2:** Hypertension control status and medication intake of the participants.

Variables	n			%
	**Puskesmas Junrejo**	**Puskesmas Batu**	**Total**	
**Hypertension control status**				
Controlled	21	51	72	27.8
Not controlled	98	95	193	72.2
**Medication**				
Antihypertensive drug	119	146	256	100

### Lifestyle factors

Table 3 shows the lifestyle factors of the participants. A total of 122 participants demonstrated a low PA level (46%). Most participants had good sleep quality (80%). Two hundred fifty-four participants had low caloric intake (95.5%). The majority had neutral food habits (55.5%) and low emotional eating (93.2%).

**Table 3:** Lifestyle factors of the participants.

Variables	n	%
**Physical activity level**		
Low	122	46
Moderate	78	29.4
Vigorous	65	24.6
**Stress level**		
Normal	251	94.7
Mild	9	4.2
Moderate	2	0.75
Severe	3	1.1
**Sleep quality**		
Good	212	80
Poor	53	20
**Caloric intake**		
Low	254	95.5
Normal	8	3.0
Excessive	3	1.1
**Food habit**		
Unhealthy	4	1.3
Neutral	166	55.5
Healthy	95	31.8
**Emotional eating**		
Low	248	93.2
Moderate	7	2.6
High	10	3.8

### Factors associated with hypertension control

The association between the lifestyle factors and hypertension control was evaluated using stepwise binary logistic regression, followed by the construction of the predictive model. The omnibus test showed that the χ2 value (25.592) was greater than the χ2 table value (21.02) (P<0.05). The Hosmer–Lemeshow test revealed that the χ2 value (10.33) was lower than the χ2 table value (19.67) (P>0.05). Therefore, the model was accepted.

The independent variables explained 13.3% of the variation in the dependent variable. The study model had an accuracy rate of 74.7%. Table 4 shows that there were five independent variables that significantly influenced hypertension control (P<0.05). These variables included sex (P=0.030), age (P=0.018), PA level (P=0.011), sleep quality (P=0.032) and stress level (P=0.030).

**Table 4:** Binary logistic regression analysis of the factors significantly associated with hypertension control.

Variables	B	Standard Error	Wald	Degree of freedom	P-value
Sex	0.832	0.383	4.713	1	0.030^*^
Age	-0.547	0.231	5.588	1	0.018^*^
Physical activity level	0.471	0.182	6.723	1	0.011^*^
Sleep quality	0.934	0.435	4.618	1	0.032^*^
Stress level	-0.808	0.371	4.731	1	0.030^*^
**Intercept**	0.307				

* p<0.05

The model formed was as follows:


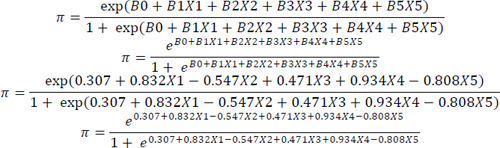


Further analysis was conducted to evaluate the influence of each category of each variable on hypertension control. It was found that moderate and vigorous PA levels were associated with hypertension control, as displayed in Table 5.

**Table 5:** Influence of the significant lifestyle factors on hypertension control.

Variables	P-value	Odds Ratio	95% confidence interval	
			**Lower**	**Upper**
**Sex**				
Female	0.090	1.945	0.927	4.360
Male		1.000		
**Age, year**				
36-45	0.190	0.209	0.010	1.643
46-55	0.105	0.409	0.138	1.215
56-65	0.350	0.641	0.255	1.668
>65		1.000		
**Physical activity level**				
Vigorous	0.042^*^	2.188	1.026	4.678
Moderate	0.000^**^	5.868	3.024	11.798
Low		1.000		
**Sleep quality**				
Good	0.081	2.264	0.950	6.100
Poor		1.000		
**Stress level**				
Normal	0.075	0.088	0.003	1.166
Mild	0.147	0.098	0.003	1.988
Severe		1.000		

* p<0.05

** p<0.01

In the initial analysis, the PA level tended to increase the probability of hypertension control by 1.602-fold. After the analysis with the inclusion of the category of each variable, moderate and vigorous PA levels influenced the successful control of hypertension by 5.868- and 2.188-fold, respectively.

## Discussion

In this study, the participants were primarily categorised as having uncontrolled hypertension (72.2%). This is in line with another report that 63% of patients with hypertension in Indonesia who receive medication still have uncontrolled hypertension.^17^ Low drug compliance; the type, dosage and combination of medications used; and an unhealthy lifestyle are some of the factors that could account for this outcome.^18^

The present study showed that 46.2% of the patients had a low PA level. The low motivation about the importance of PA among patients might explain this phenomenon. Islam and colleagues reported in their study that positive attitudes towards PA among patients with hypertension were not directly proportional to actions.^19^ Another possible explanation for the low PA level among patients is inadequate health programme implementation or community coverage.^20^

Based on our results, the PA level tended to increase the probability of hypertension control by 5.868- (moderate PA) and 2.188- fold (vigorous PA). This finding is supported by the report of Alsairafi that explained that there was an increased risk of uncontrolled hypertension in individuals who were not physically active.^21^ A randomised controlled trial among 237 patients with hypertension given a PA intervention for 9 months found a decrease in systolic BP and an increase in the proportion of respondents with controlled BP.^22^ Exercise in patients with hypertension could reduce systolic and diastolic BPs by 5–7 mmHg.^23^ This finding is in accordance with WHO recommendations to routinely perform moderate-intensity PA of at least 150 min a week, vigorous-intensity PA of at least 75 min a week or any combination of moderate- and vigorous-intensity PA.^24^ Vigorous-intensity PA among older adults, the predominant participant age categorisation in this study, was associated with an acute and transient increase in the risk of sudden cardiac death and acute myocardial infarction.^25^ Moreover, older adults who engaged in vigorous-intensity PA were more likely to fall.^26^ Therefore, older adults with hypertension must be cautious if they plan to perform vigorous-intensity PA. Additionally, medical teams should educate patients about the risk of falls, the side effects of vigorous-intensity PA and ways to monitor their vital signs if they choose to engage in such PA.

The regression analysis revealed that stress level tended to increase the probability of having controlled hypertension (p=0.030). This finding is supported by that of a previous study that investigated the effect of a mindfulness-based stress reduction (MBSR) programme on the outcome of patients with CVD. The study showed that the MBSR programme was effective in reducing systolic BP.^24^

The stepwise binary regression analysis showed that good sleep quality tended to increase the probability of controlling hypertension. Similarly, Ali et al.^14^ found that optimising sleep quality and sleep duration of >6 h per night improved hypertension control and was associated with changes in systolic BP within 3 months of follow-up. In addition, a study involving 33,341 respondents found that poor sleep quality was independently associated with high systolic BP and hypertension events in youth and middle-aged individuals.^9^

Another essential finding of the present study is that the women tended to have controlled hypertension compared with the men. Women have better control over their hypertension than men because they are more conscious of their health and manage their hypertension well.^27^ The fact that androgens, one of the primary male hormones, cause blood vessels to vasoconstrict is another issue.^28^

The regression analysis revealed that the older the patient, the lower the probability of controlling hypertension (0R=0.579, 95% CI=0.368–0.911). A previous study showed that older respondents (>60 years) had a 4.4 times greater chance of having uncontrolled BP than younger respondents due to the ageing process and the presence of comorbidities.^18,29^

This study has several strengths. First, the association of patient profile with hypertension control was evaluated, which has not been conducted before. Second, the study provides an indicator of programme success and emphasises the need for new strategies or revitalisation of existing programmes related to hypertension management based on the lifestyle factors. Third, the study was conducted at the Puskesmas, where most hypertension cases are routinely controlled.

Despite the strengths, this study has the following limitations. (1) The study’s response rate fell short of the minimum required for an ideal participant in this kind of research. (2) The duration was relatively insufficient. (3) The research location was restricted to Puskesmas Batu and Junrejo. (4) A cross-sectional design was adopted, which could not establish a causal relationship. (5) There was potential information bias. (6) Convenience sampling was conducted, meaning that the P-values and CIs could not be interpreted, and all estimates were subject to selection bias. (7) The AFHC, which was intended for adolescents rather than adults or older adults, was used. This questionnaire may therefore not be applicable. The interaction between smoking, adherence and the type of antihypertensive medications, which are potential confounding factors, still needs to be investigated. Therefore, the results of this study may be generalisable to specific populations only.

Future studies need to measure other lifestyle factors and the biochemical condition of patients, which also play an essential role in the successful control of hypertension. A case-control, multicentre study with a larger sample size or a mixed study about the experience and satisfaction with treatment in the Puskesmas among patients with hypertension should be conducted.

## Conclusion

Most patients with hypertension still have uncontrolled BP There is an association of age, sex, PA level, stress level and sleep quality with hypertension control. Based on the categorical analysis of each parameter, moderate and vigorous PA levels influence hypertension control.

## Data sharing statement

The raw data used in this study are available on https://doi.org/10.6084/m9.figshare.24878427.v1.

**How does this paper make a difference in general practice?**

- Lifestyle factors, mainly moderate and vigorous physical activity (PA) levels, can contribute to the management of hypertension.
- The study findings can be used to evaluate the effectiveness of the hypertension control programme implemented by the Puskesmas.
- The study provides validated evidence that can be used to plan health policies that suit the needs of patients with hypertension and focus on comorbidities and increasing PA levels.
- The results of epidemiological studies can be used to provide information and education to the public about the improvement of lifestyle factors contributing to the successful control of hypertension.
